# Oil Droplet Capture by Tunicates

**DOI:** 10.1093/iob/obaf045

**Published:** 2025-11-26

**Authors:** K Beaudry, C B Cameron

**Affiliations:** Département de sciences biologiques, Université de Montréal, Montréal, QC, H3C 3J7, Canada; Département de sciences biologiques, Université de Montréal, Montréal, QC, H3C 3J7, Canada

## Abstract

Species of filter-feeding invertebrates are exposed to natural oil droplets or petroleum oil droplets in water, and many species feed on these droplets. Here, we investigate oil droplet capture by benthic tunicates. We used videography, dissections, and tetramethylrhodamine isothiocyanate (TRITC) fluorescence microscopy to study the capture of oil droplets by 10 different species of tunicate. Eight of nine species fed on waste motor oil demonstrating that it is a general phenomenon. The exception was *Clavelina huntsmani. Corella willmeriana* fed on light crude oil based on evidence of droplets in the branchial basket, gut, and feces. These results demonstrate that tunicates can provide an entry for oils into marine food webs. A further experiment found that *Styela gibbsii* fed on emulsions of fish, canola, marine 10W-30, semi-synthetic 2-cycle, and waste 5W-20 oil in filtered seawater and unfiltered seawater. It showed no selectivity despite differences in chemistry, density, viscosity, and interfacial tensions. Finally, the size distribution of oil droplets captured by *S. gibbsii* and *Ciona intestinalis* were compared to the feeding trial emulsions and found to be significantly narrower, and on the smaller end of the range. This study provides some general insights into oil droplet capture by tunicates, the mechanics of droplet capture, the absence of selection based on the type of oil, and oil droplet size capture. Tunicates are some of the most ubiquitous and abundant animals in the world’s oceans and the pelagic species significantly alter global carbon cycles. Here, we show that benthic species, common on docks and wharves, ingest natural occurring and engine oils, offering a new puzzle piece in our knowledge on the bioaccumulation and trophic transfer of oils in marine food networks.

## Introduction

Oil droplets are ubiquitous in marine environments. They may arrive from the decay of organic tissues (e.g., fish, krill, or algae), or from petroleum seeps and spills. The ecology of petroleum oils in the marine environments is a vast subject that includes bodies of work on its immediate ecological impacts (Zhang et al. 2024), its long-term effects (Kingston 2002), ecotoxicology (Ossai et al 2024, Sahoo et al. 2024), oil behavior and droplet mechanics (Boufadel et al. 2023), and restoration and recovery (Zengel et al. 2021, Diefenderfer et al 2022). Which species are responsible for capturing these droplets, providing an entrance to the marine food web, and how these droplets are captured (or detached) are emerging topics. Animal oil droplet capture is inherent to any inquiry into oil droplet ecology. It is similar to the better-known process of particle capture but is inherently more complex because it is a two-fluids (and an appendage) process. Droplet capture and loss have been addressed in crustaceans that feed with external, hard, and articulated appendages, in polychaetes that feed with external ciliated radioles, but not animals that feed with an internal ciliated pharynx perforated with gill pores that includes hemichordates, cephalochordates, and tunicates.

Phylum Tunicata is comprised of sessile, benthic ascidians, or sea squirts, and free-swimming pelagic tunicates (Lemaire 2011). Tunicates are filter-feeding invertebrates that are found in abundance in Earth’s oceans (Bone et al. 2003). The name, tunicate comes from the firm, flexible, and barrel shaped body covering, called a tunic. Most tunicates live with the posterior end of the barrel attached firmly to a fixed object, and have two openings, or siphons. They filter-feed on plankton by pumping seawater into the inhalant siphon, across a branchial basket, and out the exhalant siphon. The ventral basket has an endostyle that casts a mucous net to capture food particles (Bone et al. 2003) across a broad range of particle sizes (Flood and Fiala-Medioni 1981; Petersen and Riisgård 1992; Riisgård and Larsen 1995; Petersen 2007) depending on the species (Lesser et al 1992; Jiang et al. 2008). This particle size selection is likely determined by the dimensions of the ciliated stigmata that perforate the basket or the distance between the capture cilia (Jorgensen and Goldberg 1953; Jørgensen 1966; Robbins 1984; Petersen et al. 1999; Paffenhöfer and Köster 2005). Ascidians capture particles by sieving. It is unclear if ascidians differentiate between particles based on material properties, density, hydrophobicity, or charge like many non-tunicate filter-feeders (Labarbera 1978; Gerritsen and Porter 1982; Monger et al. 1999; Rosa et al. 2013; Dadon-Pilosof et al. 2017; Rosa et al. 2017). Even less is known about oil droplet capture in ascidians.

The traditional dimensionless number used to characterize flow in fluid dynamic is the Reynolds number. Our definition of Re is based on sieving, the capture of a particle between two cylindrical fibers. Particle capture through sieving is the most familiar kind of biological filter. It is how cilia capture particles, where particles greater than inter-fiber distance *L* can only be caught by the filter. Particle capture through sieving is characterized by the inter-fiber distance *L* and a Reynolds number that is obtained by the following equation:


\begin{eqnarray*}
{\mathop{\mathrm{Re}}} = \frac{{{\rho }_{\mathrm{w}}{U}_{\mathrm{w}}(2{R}_{\mathrm{f}})}}{{{\mu }_{\mathrm{w}}}},\end{eqnarray*}

where ρ_w_ is the water density, *U*_w_ is the stream velocity, *R*_f_ is the radius of the fiber, and μ_w_ is the viscosity of the water (Mehrabian et al. 2018). The majority of filter feeders feed in an environment where the Reynolds number is of the order of 10^−5^–10^2^ (Koehl 1993). A tunicate cilium operates at the lower end because of the small size of cilia.

Droplet size, viscosity, and density determine capture in copepods, barnacles, and polychaete worms (Letendre et al. 2020; Beaudry and Cameron 2024). Surfactants, the chemicals used to disperse oil during spills, also play an important role in droplet capture and loss by *Daphnia* and copepods (Almeda et al. 2016; Letendre et al. 2023). Lee et al. (2012) found that the rate of oil droplets captured increases with increased droplet concentration by the pelagic doliolid tunicate *Dolioletta gehenbauri.* While large oil droplets are more buoyant and quicker to rise toward the surface, small oil droplets move more slowly, which will increase their time in the water column, and increase their availability for capture by filter feeders (Mehrabian et al. 2018). As such, droplets captured by tunicates should preferentially be the smaller ones in an oil emulsion.

Here, we investigate oil droplet capture by benthic tunicates. The objectives of this study are (1) to determine if nine species will capture and ingest droplets of motor oil, (2) to determine if a 10th species, *Corella willmeriana*, uses the branchial basket to capture crude oil, (3) to know if *Styela gibbsii* will ingest canola oil, fish oil, and three types of motor oil, and to (4) quantify the oil droplets size ranges captured by *S. gibbsii* and *Ciona intestinalis.*

## Materials and methods

Ten species of tunicate were collected for these studies. Five Pacific species were collected from the dock at the Bamfield Marine Sciences Centre, British Columbia, Canada during July 2023; three Atlantic species were collected from the dock at the Darling Marine Centre, Damariscotta River, Maine during May 2023; and two Pacific species were collected from docks in Victoria Harbour, British Columbia by WestWind SeaLab during fall 2023 (Table 1). All species were transported to the Université de Montréal for experimentation. These species were chosen because they live at shallow depths, and on docks where they are exposed to boat motor oil and petroleum. They included solitary and social species from subtidal and intertidal rocks and floating docks. Animals were maintained in aquaria where they fed once a week with a commercial blend of a commercial blend of unicellular *Tetraselmis* sp. (Brightwell Aquatics 10–15 micron PhytoGreen-M). The animals were tested for oil droplet capture within 4 weeks of capture. All experiments were done at 12°C.

**Table 1:** Information on the investigated tunicate species (Kozloff 1987)

Species	Order: Family	Type	Sampling site	Habitat
*Boltenia villosa* (Stimpson, 1864)	Stolidobranchia: Pyuridae	Solitary	Bamfield Inlet (Barkley Sound, Pacific, Canada)	Hard bottom
*Ciona intestinalis* (Linnaeus, 1767)	Phlebobranchia: Cionidae	Solitary	Damariscotta River (Northwest Atlantic, USA)	Floating docks
*Clavelina huntsmani* (Van Name, 1931)	Aplousobranchia; Clavelinidae	Social	Bamfield Inlet (Barkley Sound, Pacific, Canada)	Hard bottom
*Cnemidocarpa finmarkiensis* (Kiaer, 1893)	Stolidobranchia: Styelidae	Solitary	Bamfield Inlet (Barkley Sound, Pacific, Canada)	Floating docks
*Corella inflata* (Huntsman, 1912)	Phlebobranchia: Corellidae	Solitary	Bamfield Marine Sciences Centre (Pacific, Canada)	Floating docks
*Corella willmeriana* (Herdman, 1898)	Phlebobranchia: Corellidae	Solitary	Victoria Harbour (Pacific, Canada)	Floating docks
*Halocynthia igaboja* (Oka, 1906)	Stolidobranchia: Pyuridae	Solitary	Bamfield Marine Sciences Centre (Pacific, Canada)	Hard bottom
*Molgula manhattensis* (De Kay, 1843)	Stolidobranchia: Molgulidae	Solitary	Darline Marine Centre (Damariscotta River, Maine)	Hard bottom
*Styela clava* (Herdman, 1881)	Stolidobranchia: Styelidae	Solitary	Darline Marine Centre (Damariscotta River, Maine)	Floating docks
*Styela gibbsii* (Stimpson, 1864)	Stolidobranchia: Styelidae	Solitary	Victoria Harbour (Pacific, Canada)	Hard bottom

### Is oil droplet capture widespread among tunicates?

**Fig. 1:**
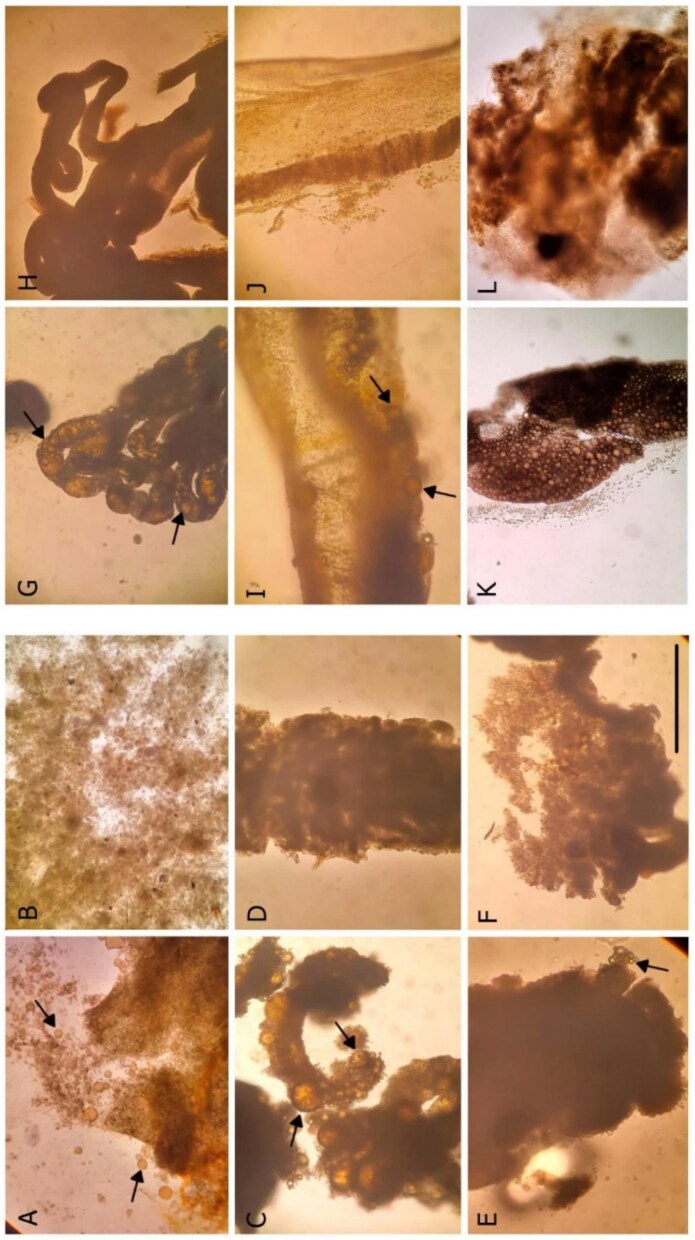
Fecal matter of tunicates after feeding on a waste motor oil-in-water emulsion. (A, B) *Molgula manhattensis*, (C, D) *Cn. finmarkiensis*, (E, F) *B. villosa*, (G, H) *H. igaboja*, (I, J) *Co. inflata*, and (K, L) *Styela clava*. In each pair the left image (A, C, E, G, I, K) is fecal matter with oil droplets, and the right images (B, D, F, H, J, L) are controls from animals not exposed to oil. Arrows point to a sample of oil droplets. Scale bar: 200 μm.

**Fig. 2:**
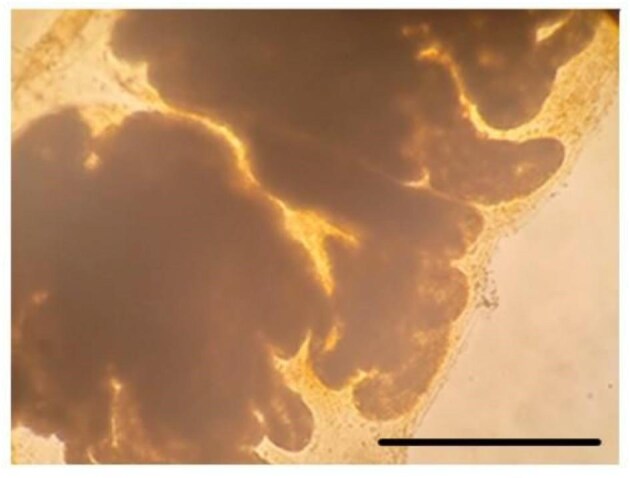
No oil was found in the fecal matter of *Cl. huntsmani* after feeding trials of waste motor oil-in-water emulsion. Scale bar: 200 μm.

**Fig. 3:**
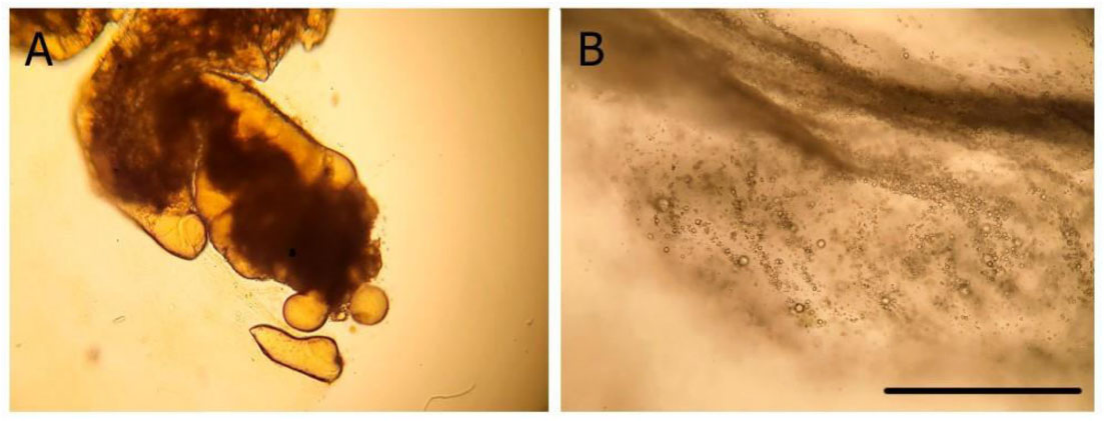
Oil droplets in the fecal matter (A) and rejected mucus (B) of *Ci. intestinalis* following feeding on a waste oil in seawater emulsion. Scale bar: 200 μm.

In the first experiment, a survey of the capture of waste 5W-20 motor oil droplets amongst tunicate was conducted with *Molgula manhattensis, Cnemidocarpa finmarkiensis, Boltenia villosa, Halocynthia igaboja, Corella inflata, S. clava* (Fig. 1), *Clavelina huntsmani* (Fig. 2), *Ci. intestinalis* (Fig. 3), and *S. gibbsii* (Movie 1). We used all of the large, solitary species available so that we could know if oil droplet capture was widespread. Feeding was determined by viewing droplets enter the atrial siphons, and ingestion was confirmed from oil drops in the fecal matters. This experiment was repeated with two individuals of each species to verify the replicability of the results. Before the motor oil emulsion feeding trials began, animals were starved for 24 h in artificial saltwater or filtered seawater to ensure a clear digestive tract and to facilitate oil observations. An individual was then placed in a motor oil-in-water emulsion (at a concentration of 65 µL per liter) for 24 h. *Ciona intestinalis* were also fed oil droplets at a concentration of 30 µL per liter. Oil feeding was determined by observation of oil droplets in the fecal pellets using an Olympus SZX16 light microscope mounted with a Samsung S21 camera at 60 frames per second. We chose 5W-20 motor oil because of its prevalence in boats and harbors where the tunicates occur.

The fluid properties of the oils and saltwater used in this study are provided in Table 2. The oil emulsions were prepared by mixing at 900 rpm for 20 min on a stirring plate using a magnetic stirrer. The emulsion was then poured into a 1-L yoghurt container where an individual tunicate was placed.

**Table 2:** The fluid properties of the oils and saltwater used in this study

Oil	Density (kg/m^3^)	Viscosity (mPas) at 10°C	Interfacial tension (mN/m)
Crude	855	98	27.1
Fish	902	40	10.1
Canola	950	121	11.4
Marine 10W-30	838	156	25.1
Semi-synthetic 2-cycle	831	92	17.5
Waste 5W-20	813	107	27.1
Seawater	1024	1.33	–

### Are oil droplets captured on the branchial basket of *Co. willmeriana?*

It was not possible to directly observe oil droplet capture by the branchial basket and mucous net because these structures are located inside of the tunicate body. *Ciona willmeriana* was used to understand the mechanics of oil droplet capture by dissection. To verify that oil was captured by the branchial basket and transported to the gut before defecation in fecal matter, four individuals of the 10th species, *Co. willmeriana*, were starved in filtered seawater for 24 h, then put in a light crude oil-in-water emulsion for 24 h. The crude oil emulsion was prepared using the same procedure as the previous motor oil feeding trials, except that it was prepared at 75 μL of oil per liter of saltwater. In other trials, crude oil was avoided because of its extreme volatile and toxic nature, but here it was used because it is auto-fluorescent under a Tetramethylrhodamine isothiocyanate (TRITC) filter, making it easy to identify and measure. After the feeding period, the four individuals were dissected and samples from the branchial basket, gut, and fecal pellets were mounted on slides and photographed under fluorescence microscopy using a TRITC filter mounted on an Olympus BX51 fluorescence microscope equipped with a Teledyne Lumenera Infinity 3 camera.

### Does *S. gibbsii* feed on various types of oils?

To determine if oil droplet capture depended on the type of oil, emulsions of fish, canola, marine 10W-30, semi-synthetic 2-cycle oils, and waste 5W-20 oil in filtered seawater, as well as waste oil in unfiltered seawater (Table 2), were each fed to seven individuals *S. gibbsii* (i.e., 6 different emulsions to 7 individuals, or 42 trials). These oils were chosen because they represent a broad range of densities, viscosities, and interfacial tensions (Table 2). These, and droplet size, are the most important variables that determine particle capture. *Styela gibbsii* was chosen for these feeding trials because it survived well in aquariums and were largely undisturbed by movements and manipulations compared to the other species. We found it to be a model species for feeding trials. Due to the internal nature of droplet capture, we could not measure the particle size range captured by the pharyngeal basket. Instead, we observed oil droplets enter the inhalant siphon then verified that oil was in the feces. The concentration of oils was 65 μL per liter of saltwater. Photos of the oil droplets in fecal matter were taken with a Samsung S21 camera mounted on an SX16 Olympus microscope.

### What size of oil droplets are captured by *S. gibbsii* and *Ci. intestinalis*?

Canola oil and waste 5W-20 motor oil was used to determine the minimum, maximum, and average droplet size distribution in the feeding trial emulsion versus the droplet size distribution that was inhaled into the siphon of one *S. gibbsii* (canola oil) and one *Ci. intestinalis* (motor oil). Oil droplet sizes were recorded within the first 2 min of these feeding trials. Motor oil was used for the initial test then to avoid using a pollutant in our animal care facility, we switched to canola. In addition to *S. gibbsii, Ci. intestinalis* were chosen for this oil droplet capture experiment because its solid particle size capture range is known. This allowed us to directly compare oil droplet size capture to particle size capture. As no droplets were seen exiting the exhalant siphon, we know that the entire size range was captured. ImageJ and its “Analyze particles” feature (Schneider et al. 2012

https://imagej.net/ij/download.html) were used to calculate the area of round shapes on images taken from videos of the capture using a set scale, when possible, and calculated the remainder by hand. A slightly different technique was used to measure the droplets sizes in the emulsions: photos were taken of the emulsion with the Teledyne Lumenera Infinity 3 camera, then the particle sizes were measured with ImageJ. Using Rstudio, the normality of the distributions was confirmed or denied with a Shapiro–Wilk test, then the oil droplet size distributions were compared to determine if they were significantly different with a Mann–Whitney U test using R.

## Results

### Is oil droplet capture widespread among tunicates?

Feeding was determined by viewing droplets enter the atrial siphons, and ingestion was confirmed from waste 5W-20 motor oil drops in the fecal matters of 8 of 9 species tested. This suggests that waste motor oil feeding may be widespread in benthic tunicates. We observed motor oil droplets in the feces of *M. manhattensis, Cn. finmarkiensis, B. villosa, H. igaboja, Co. inflata, Styela clava* (Fig. 1), *Ci. intestinalis* (Fig. 3) but not *Cl. huntsmani* (Fig. 2). Oil droplets in feces appears translucent and spherical (Figs. 1–3). The control trials of individuals, which were fed on a diet that lacked oil droplets, had no oil in the feces (Figs. 1–3). Video of the inhalation of droplets into the atrial siphon of the nineth species, *S. gibbsii* (Movie 1), demonstrate that they actively inhale waste motor oil droplets. *Clavelina huntsman* was unique in that it did not inhale motor oil droplets at neither 65 μL nor 30 μL per liter of seawater. No inhalant current was observed in the emulsion, which we interpret as ciliary arrest. Its feces lacked oil droplets (Fig. 2). An additional third feeding trial was done before trials were terminated.

*Ciona intestinalis* did not feed at a concentration of 65 μL per liter for 24 h, so we lowered the oil in seawater concentration to 30 μL per liter. At this lower concentration, *Ci. intestinalis* was found to inhale, capture, ingest into the gut, and defecate the motor oil droplets (Fig. 3A). At the higher concentration, there were no oil droplets in the fecal pellets. Instead, the mucous net full of oil droplets was ejected from the mouth (Fig. 3B). The difference in the size and shape of the droplets between the fecal pellet and the mucous shows a particularly extreme example of coalescence that can occur between droplet capture and defecation.

### Are oil droplets captured on the branchial basket of *Co. willmeriana?*

**Fig. 4:**
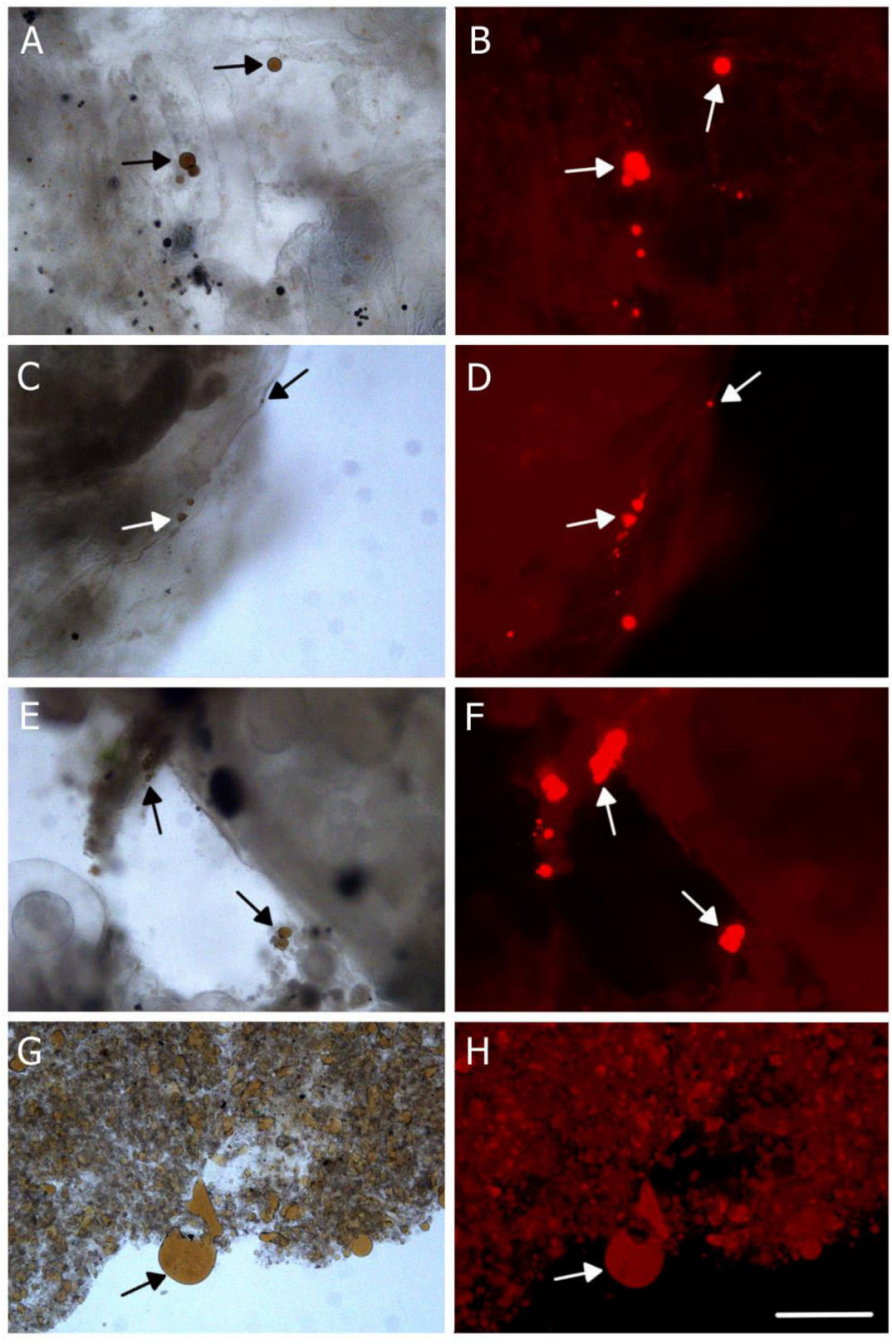
*Corella willmeriana* captured and ingested light crude oil droplets, viewed with a light (left column) and TRITC autofluorescence (right column). (A, B, C, D) Droplets captured by the branchial basket of an individual after the feeding period. (E, F) Gut content. (G, H) Fecal matter. Arrows point to a sample of oil droplets. Scale bar: 200 μm.

To verify that oil droplets were captured by the branchial basket, transported to the gut, and defecated, four individuals of a 10th species, *Co. willmeriana*, were fed a light crude oil-in-water emulsion (75 μL of oil per 1 L of saltwater). After feeding, a dissection of the individual specimens was done. The branchial basket with its mucous net was removed and observed to have abundant oil droplets (Figs. 4A–D). Then, the gut was opened to reveal oil droplets (Fig. 4E and F). Finally, oil droplets were observed in the fecal matter (Fig. 4G and H). These results suggest that the mechanism of oil droplet capture, ingestion, and defecation is like particle capture.

### Does *S. gibbsii* feed on various types of oils?

**Fig. 5:**
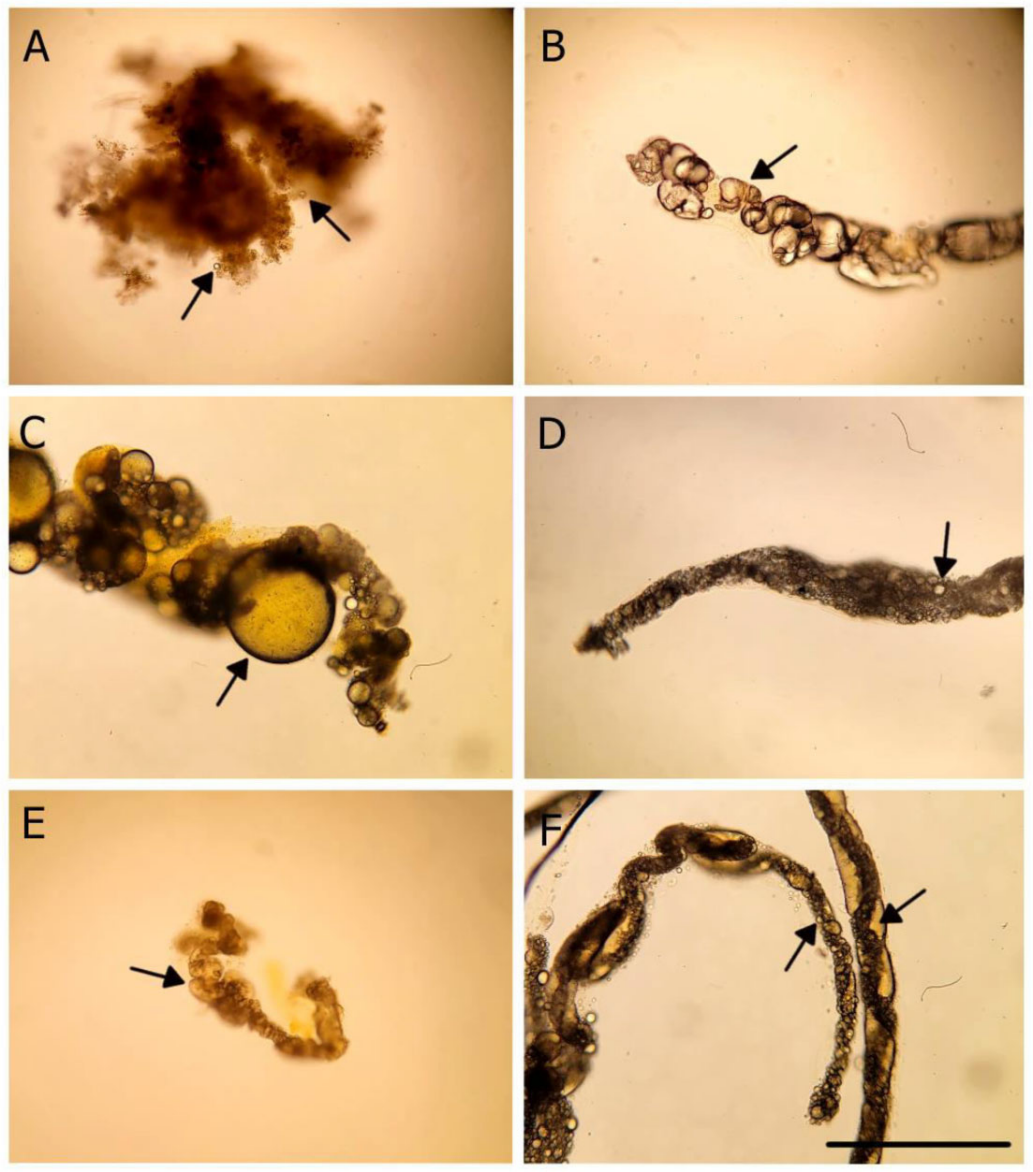
Captured and ingested oil droplets in the fecal matter of *S. gibbsii* following feeding trials. (A) Canola oil. (B) Fish oil. (C) Marine engine oil. (D) Semi-synthetic engine oil. (E) Waste oil. (F) Waste oil with unfiltered seawater. Arrows point to a sample of oil droplets. Scale bar: 200 μm.

To determine if oil droplet capture varied depending on the type of oil, *S. gibbsii* individuals were put in filtered seawater and fed fish, canola, marine 10W-30, semi-synthetic 2-cycle oils, and waste 5W-20 oil in filtered and unfiltered seawater (Fig. 5). In all 42 feeding trials, oil droplets were seen to enter in the inhalant siphon and in the fecal matter of the animals (Fig. 5). For the canola oil, few droplets were observed in the feces, suggesting that it was absorbed by the gut (Fig. 5A). Fish oil was observed in the feces (Fig. 5B). Motor oil droplets did not appreciably coalesce, and the oil remained as droplets in the feces (Fig. 5C–E). *Styela gibbsii* ingested waste oil droplets in both filtered and unfiltered seawater. A high concentration of oil was found in the feces of the unfiltered seawater treatment (Fig. 5F). These results suggest that *S. gibbsii* does not discriminate by the type of oil despite the disparity in chemical composition, density, viscosity, or interfacial tension (Table 2).

### What size of oil droplets are captured by *S. gibbsii* and *Ci. intestinalis*?

**Fig. 6:**
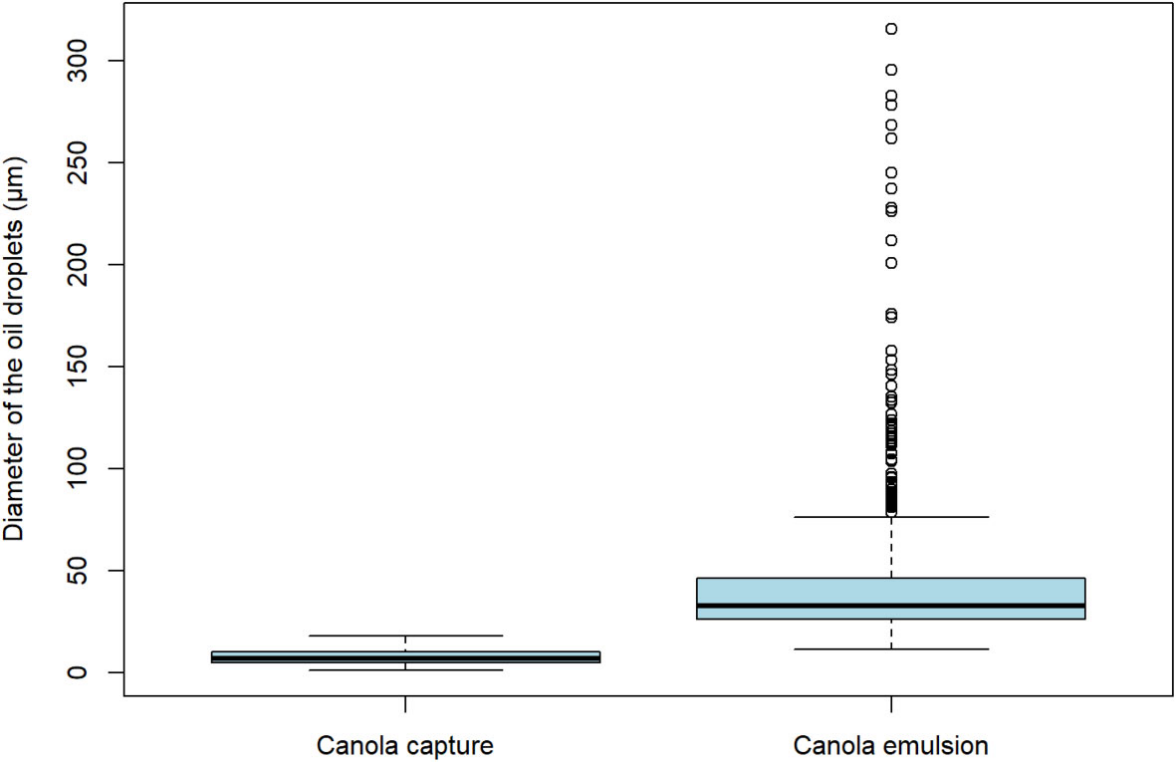
Boxplot of the distribution of canola oil droplets present in the seawater emulsion vs. those that entered the inhalant siphon of *S. gibbsii*. Canola emulsion *n* = 812, canola capture *n* = 56.

Canola oil was used to determine the minimum, maximum, and average droplet size inhaled by *S. gibbsii*, by measuring droplet sizes as they entered the inhalant siphon. The inhalant siphon diameter was variable to about 5 mm. The canola oil emulsion droplet diameter varied from 11.40 to 315.80 μm (mean 42.62 μm) (Fig. 6) and those captured by *S. gibbsii* varied from 1.11 to 17.75 μm (mean 7.89 μm). All of the inhaled droplets were retained. To determine the normality of these distributions, Shapiro–Wilk tests were conducted, and both found to be statistically significant at (*W* = 0.952, *P*-value = 0.03), rejecting the null-hypothesis of normality. Thus, a Mann–Whitney U test was performed to determine if the difference between the canola oil size distribution in the emulsion vs. the droplet sizes that entered the inhalant siphon were statistically significant, and a *W* = 186, and *P*-value of *P* < 2.2e-16 was obtained. This shows that the droplet size distributions of the emulsion and the captured droplets are significantly different.

**Fig. 7:**
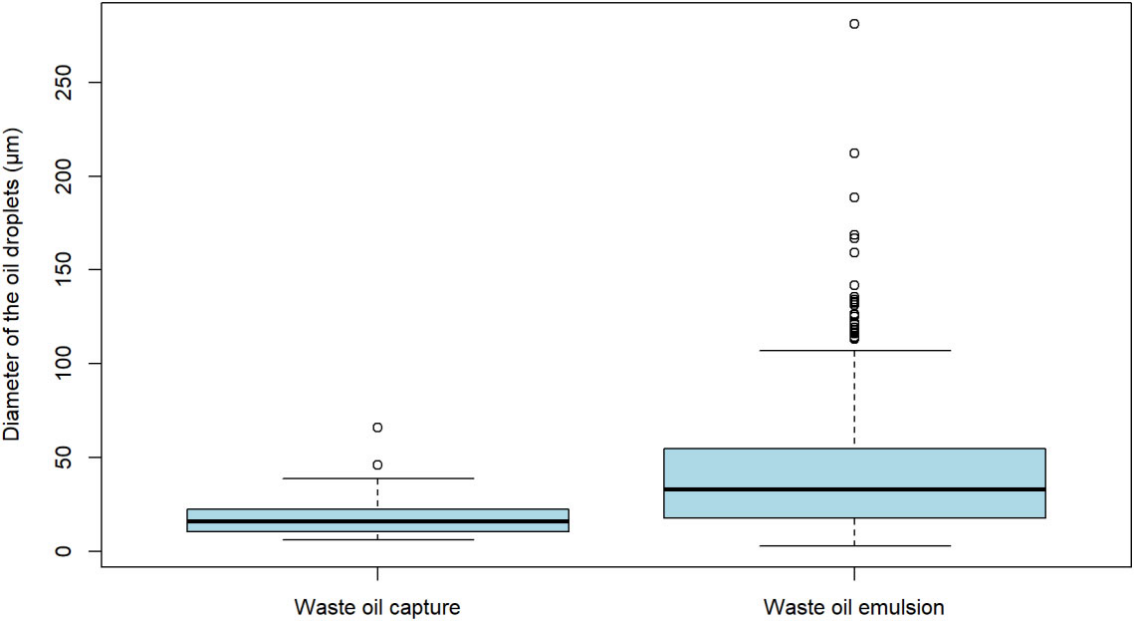
Boxplot of the distribution of waste motor oil droplets present in the seawater emulsion vs. those that entered the inhalant siphon of *Ci. intestinalis*. Waste oil emulsion *n* = 693, waste oil capture *n* = 83.

Waste 5W-20 motor oil was used to determine the minimum, maximum, and average droplet size ingested by *Ci. intestinalis.* The inhalant siphon diameter was variable to about 4 mm. The waste motor oil emulsion droplet diameter varied from 2.77 to 281.35 μm (standard deviation mean of 40.12 μm). The mean emulsion droplet diameter for the droplets captured by *Ci. intestinalis* varied from 6.2 to 65.81 μm (standard deviation mean of 18.05 μm) (Fig. 7). To determine the normality of these distributions, Shapiro–Wilk tests were conducted, and found to be statistically significant at (*W* = 0.861, *P*-value = 2.61e-07), rejecting the null-hypothesis of normality. Thus, a Mann–Whitney U test was performed to determine that the difference between the motor oil size distribution in the emulsion versus the droplet sizes that entered the inhalant siphon were statistically significant with *W* = 1472, and *P*-value = 3.54e-13 was obtained. This shows that the droplet size distributions of the emulsion and the captured droplets were significantly different.

## Discussion

The results of our first feeding trials demonstrate that waste 5W-20 motor oil drops were fed upon by 8 of 9 species tested (Figs. 1, 3, 4, and 5). This suggests that feeding on waste motor oil droplets seawater is a general phenomenon of benthic tunicates. This result compliment and extends the finding of oil droplet capture and ingestion by crustaceans (Almeda et al. 2013, Almeda et al. 2016, Letendre and Cameron 2022), sabellid and serpulid polychaete worms (Beaudry and Cameron 2024), and the pelagic doliolid tunicate *D. gehenbauri* (Lee et al. 2012). Feeding trials on the social tunicate *Cl. huntsmani* revealed that they do not ingest droplets from a waste motor oil-in-water emulsion. Instead, in the presence of motor oil droplets they seize to inhale seawater, presumably by ciliary arrest of the branchial basket cilia. The reason for this sensitivity we cannot speculate. At a waste motor oil concentration of 65 μL per liter *Ci. intestinalis* captured, then rejected the oil droplets by expelling the oil-droplet laden mucous net out of the inhalant siphon. This was achieved by a muscular contraction of the pharynx muscles, and no oil was found in the feces. At a lower concentration of 30 μL per liter it fed on the waste 5W-20 motor emulsion the feces contained droplets. The rejection behavior is a mechanism to avoid clogging and similarly occurs when too many particles accumulate in the branchial basket (Carver et al. 2006; Conley et al 2018). It is a response to the quantity of food rather than the quality (Jorgensen and Goldberg 1953, Robbins 1984, Petersen et al 1999). Compared to other tunicates, *Ci. intestinalis* is an aggressive pump (Carver et al 2006), so may have accumulated more droplets, and larger droplets, contributing to clogging of the branchial basket. Also, the oil laden mucous net may have been rejected because motor oil (and most oils) float, or cream, towards the water surface (Mehrabian et al. 2018). This droplet buoyancy may irritate the animal or limit its ability to manipulate and transport the net to the gut. The concentration of oil emulsion in seawater following an oil spill, versus oil pollution typical in boat harbors, versus in the proximity of a decaying animal may be an important factor that determines its entry into marine food webs by tunicates.

We were unable to directly observe the mechanics of oil droplet capture inside of the tunicates, so we did dissections of *Co. willmeriana* that revealed crude oil droplets in the branchial basket, gut, and feces (Fig. 4). From these observations, we think that the oil droplets were captured on the mucous net, concentrated dorsally, and transported to the gut in the same manner that particles are captured, transported to the gut, and deficated (Bone et al. 2003; Holland 2016).

We found that the tunicate *S. gibbsii* did not discriminate on the type of oil. It fed on fish, canola, marine 10W-30, semi-synthetic 2-cycle and waste 5W-20 oil in filtered seawater, and waste motor oil in unfiltered seawater (Fig. 5). Parallel experiments with flavored and untreated polystyrene spheres reveal differences in taste discrimination with some copepods, *Daphnia*, cladocerans, and rotifers (Friedman and Strickler 1975; Hartman & Hartman 1977; DeMott and Watson 1991; Kerfoot and Kirk 1991; Almeda et al. 2016). However, non-selective particle capture is also common within rotifers (DeMott 1986), demonstrating interspecific variability for the chemical composition of particles. Polychaete sabellid and serpulid worms exhibited no taste selectivity when exposed to these same types of oil droplet emulsions used in this study (Beaudry and Cameron 2024). Like oil, *Ci. intestinalis* does not distinguish between inorganic and organic particles (Robbins 1984). Petroleum (and canola) oil droplets are not typically encountered by filter-feeders, so they may lack adaptations to avoid them. The toxicity of petroleum oils to ecosystems is well documented (Fuller et al. 2004; Couillard et al. 2005; Almeda et al. 2013; Buskey et al. 2016) and the inability to discern them from naturally occurring and nutritious oils could exacerbate their impacts on marine ecosystems.

Unlike solid particles, oil droplet sizes may decrease in an emulsion, from the moment of capture to ingestion and defecation, complicating efforts to quantify the distribution of particle sizes captured. Oil droplets can be cleaved by feeding appendages of copepods (Uttieri et al. 2019), and a similar phenomenon may occur when droplets were impacted by branchial basket cilia. Similarly, oil droplet size may increase in an emulsion, from the moment of capture to ingestion and defecation. *Corella willmeriana* captured and ingested light crude oil droplets that were larger than the largest size in the emulsion (Fig. 4G and H) indicating coalescence or Ostwald ripening (Mehrabian et al. 2018) occurred somewhere between the branchial basket and defecation. For these reasons, and that capture is hidden inside of the pharynx, it is exceedingly difficult to determine size selection of oil droplets by the tunicate branchial basket. Instead, we sought to determine if there was a difference in oil droplet size distribution between the experimental feeding trial emulsion and oil droplets that were measured entering the inhalant siphon of tunicates *S. gibbsii* and *Ci. intestinalis*. In these species, the inhaled size ranges were significantly narrower, and on the smaller end of the range of distributions compared to their respective emulsions. We think this was due to gravitational creaming, whereby larger droplets were more buoyant, making them less available for capture over time (Beaudry and Cameron 2024). The size range of canola oil droplets captured by *S. gibbsii* were 1.11–17.75 μm in diameter (Fig. 6) and the size range of waste motor oil capture by *Ci. intestinalis* were 6.20–65.81 μm (Fig. 7). Paradoxically, the smallest droplets inhaled were smaller than the smallest in the emulsion. This may have been due to droplet fission that occurs with a change in surface tension, or due to increased dissolution as droplets accelerated toward the incurrent siphon. The range of particle capture and mesh size of *S. gibbsi* is unknown, but they will consume nauplius and veliger larvae (Barnes and Barnes 1965; Bingham and Walters 1989; Strathmann et al 1993). The mesh size of *Ci. intestinalis* is 0.6 μm by 0.4 μm (Flood and Fiala-Medioni 1981; Holley 1986). Its optimal particle feeding size range is between 1 and 10 μm (Petersen and Riisgård 1992), with particles as small as 1 μm retained with 70% accuracy, and larger with 100% retention (Randlov and Riisgård 1979, Jorgensen et al. 1984). Whereas some authors suggest that *Ci. intestinalis* do not sort solid particles by size (Jorgensen and Goldberg 1953, Robbins 1984, Petersen et al. 1999), others state that tunicates can discriminate between particles of different sizes by adjusting their retention efficiency (Lesser et al. 1992; Jiang et al. 2008). Our experiments on oil droplet size capture did not determine an upper or a lower size, and we cannot say if retention efficiency changes with droplets less than 6.2 μm or greater than 65.8 μm. In this range, 100% of the droplets are retained.

This study shows that the capture of waste motor oil droplets by benthic tunicates common to harbors is a general phenomenon, that the mechanism of capture is like that for solid particles, and that tunicates may not discriminate feeding on a variety of different oils. Ours is the first attempt to characterize oil droplet size capture, which differs from the experimental emulsion size distribution. This difference though is due to the mechanics of a fluid (oil) in a fluid (seawater) rather than the nature of the capture mechanism that characterize particle capture studies. Oil emulsions are dynamic and even more so in the presence of feeding animals. With this knowledge in hand, we are curious to understand the precise mechanics of oil droplet capture by a tunicate mucous mesh. How does it differ from solid feeding appendages and ciliated radioles of polychaetes? Future studies may aim to determine the role of flow in droplet capture. Our feeding trials were done in the absence of flow at very low Reynolds numbers. *Ciona intestinalis* functions at low Reynolds numbers (Conley et al. 2018) but its external, environmental flow can be great, which is likely to alter droplet availability by making larger droplets more available. Natural and man-made surfactants alter the stability of emulsions (Letendre et al. 2023), and so might be expected to alter capture success, especially if the droplets resist deformation. In this case, the mechanics of their capture may approach solid particles, including microplastics (Kremser and Schnug 2002; Howarth 2008; do Sul and Costa 2014; Rogers et al. 2020). This discovery of oil droplet consumption by tunicates refine the critical analyses of how oils enter, persist, degrade, and transfer through marine trophic networks.

## Supplementary Material

obaf045_Supplemental_Files

## Data Availability

Not applicable.
